# Translation elongation inhibitors stabilize select short-lived transcripts

**DOI:** 10.1261/rna.080138.124

**Published:** 2024-12

**Authors:** Nicolle A. Rosa-Mercado, Allen R. Buskirk, Rachel Green

**Affiliations:** 1 Johns Hopkins University School of Medicine, Department of Molecular Biology & Genetics, Baltimore, Maryland 21205, USA; 2 Howard Hughes Medical Institute, Johns Hopkins University School of Medicine, Baltimore, Maryland 21205, USA

**Keywords:** translation elongation, mRNA stability, codon optimality, ribosome collisions

## Abstract

Translation elongation inhibitors are commonly used to study different cellular processes. Yet, their specific impact on transcription and mRNA decay has not been thoroughly assessed. Here, we use TimeLapse sequencing to investigate how translational stress impacts mRNA dynamics in human cells. Our results reveal that a distinct group of transcripts is stabilized in response to the translation elongation inhibitor emetine. These stabilized mRNAs are short-lived at steady state, and many of them encode C2H2 zinc finger proteins. The codon usage of these stabilized transcripts is suboptimal compared to other expressed transcripts, including other short-lived mRNAs that are not stabilized after emetine treatment. Finally, we show that stabilization of these transcripts is independent of ribosome quality control factors and signaling pathways activated by ribosome collisions. Our data describe a group of short-lived transcripts whose degradation is particularly sensitive to the inhibition of translation elongation.

## INTRODUCTION

Translation elongation inhibitors are used to study a wide range of cellular behavior. These inhibitors are commonly used to arrest translation, for example, to rule out a role for novel protein synthesis during stress responses and to induce signaling cascades in the ribotoxic stress response (RSR). Although these inhibitors are often used, it is not well understood how they affect global transcription and mRNA decay, and these effects on mRNA dynamics could affect the interpretation of data generated after inhibition. Therefore, we set out to understand the impact of translation elongation inhibitors on the transcriptome.

mRNA decay is known to be tightly coupled to translation (Hu et al. 2009; Roy and Jacobson 2013; Pelechano et al. 2015). After an mRNA is deadenylated, it can be degraded by the exonuclease Xrn1 in the 5′ to 3′ direction or by the cytoplasmic exosome in the 3′ to 5′ direction. Work in yeast has demonstrated that exonucleolytic degradation of mRNAs from the 5′ end can begin with ribosomes still translating the message (Hu et al. 2009). Further evidence for direct connections between mRNA degradation and translation is found in the observation that codon usage heavily influences mRNA stability: transcripts enriched in slowly decoded codons have shorter half-lives than other transcripts (Presnyak et al. 2015; Narula et al. 2019; Wu et al. 2019; Forrest et al. 2020). Multiple studies established that slow elongation on transcripts enriched in suboptimal codons triggers cotranslational mRNA decay (Presnyak et al. 2015; Narula et al. 2019; Wu et al. 2019; Forrest et al. 2020) through a mechanism known as codon optimality-mediated decay (COMD) (Buschauer et al. 2020).

Other examples of a tight connection between mRNA stability and translation are mRNA surveillance pathways that target problematic mRNAs (Harigaya and Parker 2010; D'Orazio and Green 2021). There are several pathways that target distinct mRNA transcripts: transcripts with premature stop codons are degraded through nonsense-mediated decay (NMD), transcripts that completely lack stop codons are degraded through nonstop decay (NSD), while mRNAs containing sequences that induce translation elongation stalls are degraded through no go decay (NGD). Each of these mRNA decay pathways is triggered by the recognition of defective translation events, highlighting again the intricate ties between translation and mRNA stability.

Generalized defects in translation have commonly been studied using translation elongation inhibitors. In recent work, these inhibitors have been used to study ribosome-activated signaling pathways that lead to broad transcriptome changes (Sinha et al. 2020). Although emetine and anisomycin work by different mechanisms, inhibiting translocation and disrupting aminoacyl tRNA binding, respectively (Dmitriev et al. 2020), both drugs induce ribosome collisions at low doses while at high doses, they simply result in ribosome stalling (Simms et al. 2017; Juszkiewicz et al. 2020; Sinha et al. 2020; Wu et al. 2020). Ribosome collisions recruit kinases that activate at least two signaling pathways (Iordanov et al. 1997; Sinha et al. 2020; Vind et al. 2020; Wu et al. 2020; Stoneley et al. 2022). The mitogen-activated protein kinase ZAKα activates p38 and JNK, which mediate the RSR and dictate cellular fate (Iordanov et al. 1997; Vind et al. 2020; Wu et al. 2020). Additionally, upon certain translational stress conditions, the integrated stress response (ISR) is activated through the kinase GCN2 (Deng et al. 2002; Wu et al. 2020). This kinase phosphorylates the initiation factor eIF2α, resulting in a widespread translation initiation block (Pakos-Zebrucka et al. 2016). These signaling pathways result in the activation of transcription factors, including c-JUN and ATF4, which reshape the transcriptome and dictate cellular fate. However, the dynamic effects to the transcriptome that result from activation of these signaling pathways have not been thoroughly assessed.

The ribosome collision interface provides a unique structure recognized by multiple quality control (QC) factors that can orchestrate the activation of transcriptional responses and impact the translatome. EDF1, for example, mediates downstream transcriptional activation of stress-responsive genes, like the proapoptotic oncogene, c-Jun (Sinha et al. 2020) and recruits the translational repressor GIGYF2 (Juszkiewicz et al. 2020; Sinha et al. 2020) that prevents translation of problematic transcripts (Hickey et al. 2020). In response to ribosome collisions, the E3 ubiquitin ligase ZNF598 ubiquitylates the ribosomal proteins eS10 and uS10 (Brandman et al. 2012; Letzring et al. 2013; Sundaramoorthy et al. 2017; Juszkiewicz et al. 2018), triggering splitting and recycling of ribosomal subunits (Matsuo et al. 2017; Hashimoto et al. 2020). Moreover, in *Saccharomyces cerevisiae* and *Escherichia coli*, the collision interface recruits the endonucleases Cue2 and SmrB, respectively, which cleave the mRNA and mediate NGD (D'Orazio et al. 2019; Saito et al. 2022). Together, these findings point toward the importance of the translation state in shaping the transcriptome through the regulation of both transcription and mRNA decay.

Here, we test the influence of translation elongation inhibition on the human transcriptome using the elongation inhibitor emetine. We used emetine at a low dose, which induces ribosome collisions and modestly activates the RSR without activating other stress response pathways like the ISR (Stoneley et al. 2022). Using TimeLapse sequencing (TL-seq) (Schofield et al. 2018), we identified a subset of transcripts that become stabilized within 1 h of low-dose emetine treatment. Interestingly, many of the stabilized transcripts encode proteins with C2H2 zinc finger domains and are enriched in suboptimal codons. The stabilized mRNAs have short half-lives, but this alone does not fully explain emetine-induced stabilization. We tested the importance of ribosome states to the stabilization of these transcripts by treating cells with low or high doses of emetine and found that their stabilization is independent of ribosome collisions. Together, our work provides insights into the impact of elongation inhibitors on mRNA dynamics and points toward a subset of short-lived transcripts that are uniquely sensitive to translational distress.

## RESULTS

### Low doses of emetine induce changes in mRNA dynamics

**FIGURE 1.**
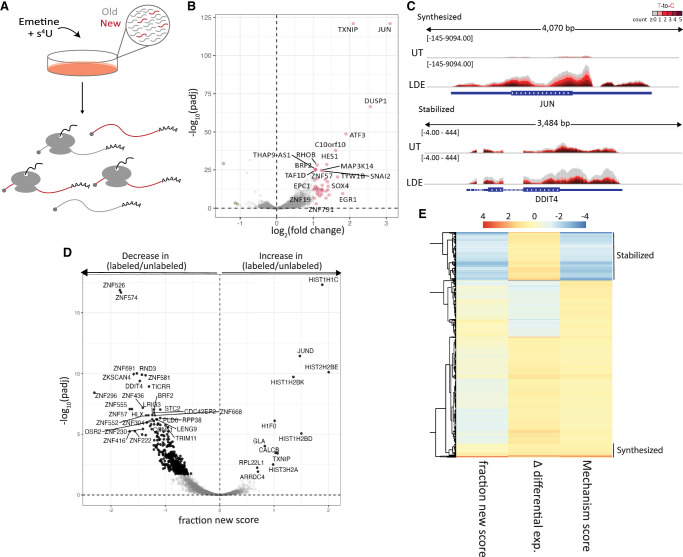
Low doses of emetine lead to changes in mRNA dynamics. (*A*) Cartoon depiction of the experimental approach. HEK-293T cells were treated with emetine and s^4^U for 1 h. Afterward, extracted RNAs were subjected to TimeLapse chemistry, cloned into libraries, and subjected to high-throughput sequencing. (*B*) Volcano plot showing differential expression analysis of TL-seq libraries from emetine-treated and untreated cells. Transcripts that increase more than twofold and have an adjusted *P*-value (*p*_adj_) of < 0.01 are shown in pink, and mRNAs that decrease in levels by more than twofold and have an adjusted *P*-value of < 0.01 are shown in dark green. (*C*) IGV browser images for a transcriptionally upregulated gene, *JUN*, and for a stabilized gene, *DDIT4*. Increasing shades of red depict an increase in the number of T-to-C conversions found in a read. (*D*) Volcano plot showing the fraction new score of expressed transcripts on the *x*-axis and the −log_10_(*p*_adj_) on the *y*-axis. (*E*) Heatmap showing bakR (Vock and Simon 2023) results obtained from TL-seq for mRNAs that are differentially expressed upon emetine treatment according to DESeq2. The *left* column shows the fraction new score calculated from changes to the T-to-C conversion ratios where orange represents an increase and blue represents a decrease. The *middle* column shows differential expression values as calculated by DESeq2, where increasing shades of orange represent upregulation and increasing shades of blue represent downregulation. Finally, the *right* column shows the mechanism score calculated by bakR, where blue indicates changes in the rate of decay and red indicates changes in the rate of synthesis driving the differential gene expression shown in the *middle* column. Example clusters of stabilized or synthesized mRNAs are labeled accordingly.

Low doses of emetine trigger changes in gene expression (Sinha et al. 2020). To determine what drives these differences, we performed TL-seq (Schofield et al. 2018) on HEK-293T cells incubated with emetine for 1 h in the presence of 4-thiouridine (s^4^U) (Fig. 1A). While high concentrations or long treatments of s^4^U impact translation and rRNA processing (Burger et al. 2013), we found that a 1 h incubation with 500 µM s^4^U yields polysome traces comparable to those obtained from untreated cells (Supplemental Fig. S1A). Additionally, labeling transcripts with s^4^U for short periods does not induce ribosome collisions, as assessed by monitoring the distribution of the collision sensor EDF1 across sucrose gradient fractions (Supplemental Fig. S1B). After metabolically labeling RNAs in HEK-293T cells with s^4^U, we performed nucleoside recoding chemistry to create apparent T-to-C conversions that can be detected through high-throughput sequencing (Herzog et al. 2017; Schofield et al. 2018). Performing metabolic labeling and emetine incubations simultaneously allowed us to distinguish between transcripts that existed prior to translation elongation inhibition and those synthesized in response to it. Using sucrose gradient sedimentation, we confirmed that low doses of emetine increased polysome abundance and induced ribosome collisions, as evidenced by the movement of EDF1 into the polysomal fractions (Supplemental Fig. S1C,D).

RNA sequencing of emetine-treated and untreated samples subjected to nucleoside recoding chemistry revealed differential expression (Fig. 1B), consistent with previous studies (Sinha et al. 2020). The levels of many transcripts increase substantially after 1 h of emetine treatment, including that of *TXNIP, DUSP1,* and *ZNF57* (Fig. 1B, highlighted in pink). We next investigated the mechanisms driving these changes by analyzing the ratio of s^4^U-labeled to unlabeled reads. As expected, samples treated with s^4^U revealed an increased number of reads containing T-to-C, but not A-to-G, conversions compared to control samples that were not incubated with s^4^U (Supplemental Fig. S1E), confirming the success of the TimeLapse chemistry.

Genome browser images of *JUN* show an increase in T-to-C conversions (shown in increasing shades of red) in emetine-treated cells compared to untreated cells, suggesting this gene is transcriptionally upregulated. On the other hand, browser images of *DDIT4* show an increase in unlabeled reads (gray) that is not accompanied by changes in the levels of labeled reads after emetine, suggesting a decrease in the rate of degradation (Fig. 1C). We used the software package bakR to analyze the ratio of T-to-C conversions (Vock and Simon 2023) to determine whether changes in gene expression were driven by changes in synthesis or decay rates transcriptome-wide. Changes in the T-to-C ratios for different mRNAs are reflected by the bakR-generated metric (bakR score) referred to here as the “fraction new score,” which represents the probability of a change in fraction new based on the ratio of labeled to unlabeled reads. Analysis of the fraction new score suggests that many transcripts experience a decrease in labeled to unlabeled reads upon emetine treatment (Fig. 1D, left side and 1E, left column). Importantly, these changes could reflect decreases in new transcription or in mRNA decay.

By comparing the fraction new score to the Δ differential expression, which reflects the DESeq2-generated *z*-score of the log_2_(fold change) of emetine-treated over untreated samples for each transcript (Love et al. 2014), we obtained a mechanism score indicating whether the changes in differential expression are most likely explained by changes in transcription or by changes in decay (Vock and Simon 2023). In Figure 1E, showing only mRNAs that are differentially expressed, increases in the fraction new score (left column) or in Δ differential expression (middle column) are shown in increasing shades of orange while decreases in the fraction new score or in Δ differential expression are shown in increasing shades of blue. Mechanism scores (right column) that are indicative of changes in transcription are shown in red and those indicative of changes in decay rates are shown in blue. As expected, low-dose emetine treatment resulted in a transcriptionally driven increase in stress response mRNAs such as *JUN* and *DUSP1* (Fig. 1 B,C; Supplemental Table S1), which have been shown to be downstream targets of the RSR (Iordanov et al. 1997; Ouyang et al. 2005; Sinha et al. 2020). However, many increases in individual transcript levels are due to decreased degradation, as exemplified by transcripts showing increasing shades of blue in both the fraction new score and the mechanism score and increasing shades of orange in the Δ differential expression (Fig. 1E).

### Select short-lived mRNAs are stabilized in response to low-dose emetine treatment

**FIGURE 2.**
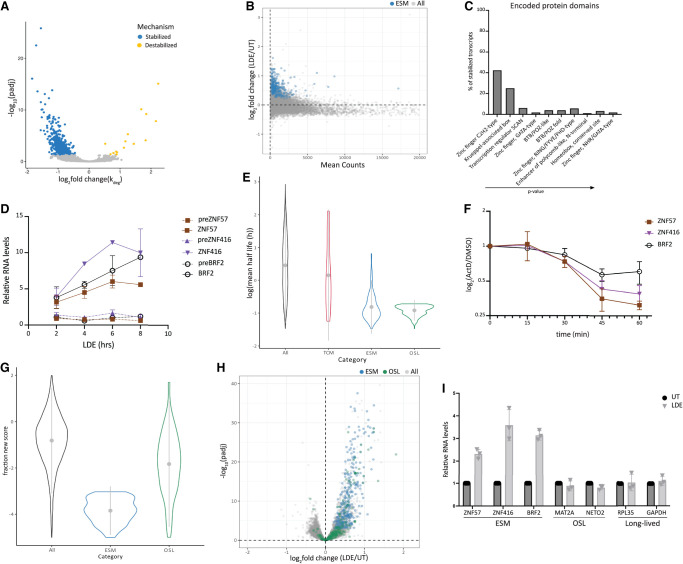
Select transcripts are stabilized in response to low doses of emetine. (*A*) Volcano plot showing the log_2_ (fold change) of the rate of degradation for all expressed transcripts as calculated by bakR on the *x*-axis and the −log_10_(adjusted *P*-value) on the *y*-axis. Stabilized mRNAs are shown in blue, and destabilized mRNAs are shown in yellow. (*B*) Minus average plot showing the mean of normalized counts obtained from DESeq2 on the *x*-axis and the log_2_(fold change) of emetine-treated cells versus untreated cells on the *y*-axis. ESMs are highlighted in blue. (*C*) Gene ontology analysis of ESMs showing enrichment of protein-encoded domains. (*D*) RT-qPCR data obtained using primers against mature mRNAs or intronic regions of representative ESMs showing that levels of these mRNAs continue to increase for up to 8 h of emetine treatment (*n* = 2). (*E*) Violin plot showing the mean half-lives calculated in K562 cells (Schofield et al. 2018) for mRNAs expressed in both K562s and HEK-293Ts (all), for mRNAs that are stabilized (ESMs), transcriptionally changed in response to emetine (TCMs), and for mRNAs that are short-lived, but not classified as ESMs (other short-lived; OSL). (*F*) RT-qPCR data were obtained from cells treated with ActD for different periods of time (*n* = 3). (*G*) Violin plot showing the fraction new score calculated using bakR on the *y*-axis for ESMs and OSLs compared to all transcripts. (*H*) Volcano plot showing the −log_10_(adjusted *P*-value) on the *y*-axis and the log_2_(fold change) of emetine versus untreated on the *x*-axis. ESMs are shown in blue, and OSLs are shown in green. (*I*) RT-qPCR for representative ESMs, OSLs (mean half-life < 1 h), and transcripts known to have a long half-life from HEK-293T cells that were treated with emetine for 2 h (*n* = 3).

Results obtained from TL-seq revealed a decreased rate of degradation rather than an increase for the majority of transcripts that undergo a change in decay upon low-dose emetine treatment (Fig. 2A). As a result of the observed decrease in decay rates, emetine-stabilized mRNAs (ESMs), which are generally expressed at low levels in untreated cells, accumulate to higher levels in emetine-treated cells (Fig. 2B, highlighted in blue). Nonetheless, low levels of expression are not sufficient to drive stabilization as many other lowly expressed transcripts remain unchanged in emetine-treated cells (Fig. 2B). Gene ontology analysis of the protein domains encoded by ESMs revealed that around 42% of these mRNAs encode proteins with C2H2 zinc finger domains (Fig. 2C). To validate these observations and further examine the temporal dynamics of the observed mRNA stabilization, we performed RT-qPCR using primers against three ESMs (*ZNF57*, *ZNF416*, and *BRF2*) targeting either the mature mRNA or intronic regions of these mRNAs. Intronic primers were used to measure changes in the transcription of these messages over time. We find that levels of these three ESMs continue to increase for up to 8 h, while transcription levels remain unchanged compared to untreated cells throughout the duration of the time course (Fig. 2D).

We reasoned that for the levels of the stabilized transcripts to increase so quickly (within 1 h of emetine treatment) without significant transcriptional changes, these mRNAs must have relatively short half-lives in untreated cells. We assessed the half-lives of ESMs in multiple ways: we used publicly available half-life calculations from K562 cells, we performed RT-qPCR after using the transcription inhibitor actinomycin D (ActD) in HEK-293T cells, and we analyzed publicly available RNA-seq data obtained from HEK-293Ts treated with ActD for different times. Half-life measurements obtained from K562 cells (Schofield et al. 2018) were used to compare the half-lives of mRNAs that we defined as ESM or transcriptionally changed (TCM) to mRNAs found in both data sets. These analyses revealed that the average half-lives of ESMs are much shorter than the average half-lives in untreated cells (Fig. 2E, ESM vs. all). To confirm that the half-life measurements calculated in K562 cells were similar to mRNA half-lives observed in HEK-293T cells, we performed RT-qPCR on certain transcripts after ActD treatment. RT-qPCR following ActD treatment clearly showed that levels of representative ESMs in HEK-293T cells decrease within 1 h of transcription inhibition (Fig. 2F). We similarly observe a decrease in levels of two TCMs (*JUN* and *DUSP1*) within 1 h of transcriptional shut-off, but long-lived transcripts whose levels do not change (*GAPDH* and *RPL35*) upon emetine treatment remain unchanged after 1 h of transcription inhibition (Supplemental Fig. S2A). Analysis of RNA-seq data from cells treated with ActD for 1, 3, or 6 h (Wu et al. 2019) similarly reveals that ESMs are short-lived compared to other expressed mRNAs (Supplemental Fig. S2B).

A critical analysis shows that not all short-lived transcripts are revealed as “stabilized” in emetine-treated cells according to the fraction new scores. We defined a set of “other short-lived mRNAs” or OSLs that have half-lives comparable to ESMs but did not meet the fraction new score or significance cutoffs to qualify as ESMs (Fig. 2G,H). Despite having similar half-lives to ESMs, the fraction new scores for OSLs reflect a lower likelihood that any increase in mRNA levels is driven by a change in the ratio of new to old reads; moreover, there is less of a decrease in the fraction new score of the OSL transcripts (green) than the ESMs (blue, Fig. 2G; Supplemental Fig. S2C). The differences in the behavior of ESMs and OSLs were confirmed with RT-qPCR: in contrast to the robust increase in representative ESM levels after 2 h of emetine treatment, we observed little or no change in the levels of mRNAs that are not ESMs and have a half-life of less than 1 h (Fig. 2I). These data suggest that while a short half-life correlates with stabilization upon emetine treatment, it is not sufficient to induce stabilization.

### Stabilized transcripts are enriched for suboptimal codons

**FIGURE 3.**
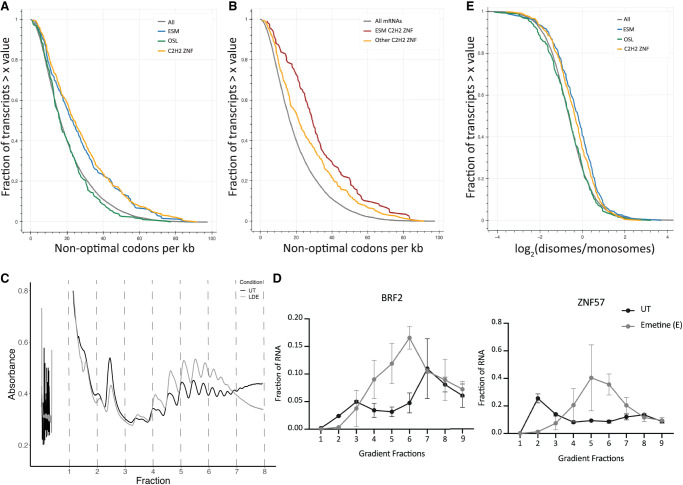
Stabilized transcripts are enriched in suboptimal codons. (*A*,*B*) Cumulative distribution function (CDF) plot showing the fraction of transcripts on the *y*-axis and the length-normalized number of suboptimal codons according to previous CSC calculations (Wu et al. 2019) on the *x*-axis for all expressed transcripts, OSLs, ESMs, and all transcripts encoding C2H2 ZNFs (*A*; *P*-value = 9.60×10^−8^ for ESMs vs. all, 0.0001 for ESMs vs. OSLs, 7.07×10^−14^ for C2H2 ZNFs vs. all), or for ESMs that are C2H2 ZNF mRNAs and other C2H2 ZNF mRNAs (*B*; *P*-value = 0.0004 for ESM ZNFs vs. OSL ZNFs, 1.29×10^−12^ for ESM ZNFs vs. all, 2.53×10^−6^ for OSL ZNF vs. all). (*C*) Polysome traces obtained from samples treated with low doses of emetine for 2 h. (*D*) RT-qPCR data for transcripts isolated from sucrose gradient fractions from cells treated with low doses of emetine for 2 h or left untreated (9 = gradient left over; *n* = 2). (*E*) CDF plot showing the log_2_(disome/monosome) for ESMs (blue), OSLs (green), C2H2 ZNF-encoding mRNAs (orange), and all expressed (gray) mRNAs on the *x*-axis and the fraction of mRNAs on the *y*-axis (ESM vs. all *P*-value = 6.76×10^−9^; ESM vs. OSL *P*-value = 0.0006; all vs. C2H2 ZNF *P*-value = 1.67×10^−6^; all vs. OSL *P*-value = 0.7060).

Since ESMs have short half-lives and codon optimality is tied to mRNA stability in eukaryotic cells (Presnyak et al. 2015; Hanson and Coller 2018; Narula et al. 2019; Wu et al. 2019; Forrest et al. 2020), we assessed the frequency of suboptimal codons in these mRNAs. Comparison of the frequency of suboptimal codons using previously calculated codon stability coefficients (CSCs) (Wu et al. 2019), a metric reflecting the correlation of codon usage and mRNA stability (Presnyak et al. 2015), showed that ESMs have a significantly higher frequency of suboptimal codons compared to other expressed mRNAs, including OSLs (Fig. 3A). C2H2 zinc finger protein-encoding mRNAs are known to be enriched in suboptimal codons (Diez et al. 2022). In our analysis, this class of transcripts exhibited a similar enrichment for suboptimal codons as ESMs, consistent with the fact that these make up a large portion of the stabilized mRNAs (Fig. 3A). However, only 29% of expressed mRNAs encoding C2H2 zinc finger proteins are stabilized in our data set. Therefore, we compared the codon optimality for stabilized C2H2 ZNF-encoding mRNAs to other expressed C2H2 ZNF-encoding mRNAs. This analysis revealed that stabilized C2H2 ZNF-encoding mRNAs are slightly more enriched in suboptimal codons than other expressed C2H2 ZNF-encoding mRNAs (Fig. 3B).

To determine if any specific codons were enriched in ESMs compared to OSLs, we assessed the abundance of each codon in these sets of mRNAs. Codon enrichments were calculated by taking the relative abundance of each codon in ESMs or OSLs divided by the relative abundance across all transcripts. These calculations demonstrate that ESMs are enriched in Cys and His codons (Supplemental Fig. S3A), in agreement with the fact that this class of transcripts includes many C2H2 ZNF mRNAs, which are not enriched in OSLs.

Due to the high frequency of suboptimal codons, we asked if knocking down subunits of the CCR4-NOT complex would impact the levels of ESMs. We performed RT-qPCR on RNAs obtained from samples where CNOT3 or CNOT4 were knocked down using siRNAs. Results from these experiments show that knocking down these factors does not impact the levels of ESMs in untreated or emetine-treated cells compared to cells transfected with a scrambled siRNA control (Supplemental Fig. S3B).

To assess the importance of codon optimality in the stabilization of ESMs, we compared the log_2_(fold change) of ESMs in emetine-treated versus untreated samples to that of mRNAs containing more than 28 suboptimal codons per kb, the top quartile in all transcripts. We find that a high frequency of suboptimal codons is not enough to elicit the emetine-induced stabilization of these mRNAs (Supplemental Fig. S3C). We next asked whether the high frequency of suboptimal codons observed for ESMs and their short half-lives were two separate features or whether they were codependent characteristics of these transcripts. To do so, we compared the mean half-lives observed for transcripts with a high frequency of suboptimal codons to that of ESM. These analyses revealed that mRNAs with more than 28 suboptimal codons per kb are not as short-lived as ESMs or OSLs (Supplemental Fig. S3D). These results demonstrate that the high frequency of suboptimal codons observed for ESMs is a feature that is independent of their short half-lives. Moreover, we compared the half-lives of ESMs encoding C2H2 ZNFs to that of mRNAs that encode these domains but are not ESMs. Similarly, these analyses show that ESM C2H2 ZNFs have shorter half-lives than non-ESM C2H2 ZNFs (Supplemental Fig. S3D).

We next asked whether there was a difference in the extent of active translation on the stabilized ESMs by investigating their polysomal distribution. To test this, we performed RT-qPCR across untreated or emetine-treated sucrose gradient polysome fractions for representative ESMs (Fig. 3C). In untreated cells, ESM *ZNF57* was predominantly found in the lighter sucrose gradient fractions, indicating that it is not heavily translated, while ESM *BRF2* was observed in the heavier sucrose gradient fractions, suggesting that it is heavily translated. However, both transcripts migrated into the polysomal fractions of the sucrose gradients upon low-dose emetine treatment, revealing that these mRNAs are both translationally active to some extent (Fig. 3D). Entrapment on polysomes was also apparent for *MAT2A*, an OSL, as well as for *GAPDH*, a long-lived mRNA whose levels are unaffected by emetine treatment (Supplemental Fig. S4A). These data show that emetine treatment generally results in the entrapment of transcripts on polysomes and that this is not a trait specific to ESMs.

To get a general sense of the translation levels for ESMs under normal conditions, we compared their translation efficiency (TE) to that of OSLs. TE values were calculated by taking the ratio of the number of reads from ribosome profiling experiments for a given transcript to the number of reads from RNA-seq (using data from Han et al. 2020). We find that the TE of ESMs was comparable to the TE of other expressed transcripts and was very slightly higher than that of OSLs (Supplemental Fig. S4B). The higher TE values may reflect ribosome pausing at suboptimal codons or higher initiation rates; these steady-state measurements do not allow us to distinguish between these possibilities.

Recent work in zebrafish has argued that mRNAs encoding zinc finger proteins are prone to ribosome collisions at steady state (Ishibashi et al. 2024). Since the ESMs are comprised of many C2H2-encoding transcripts, we reanalyzed disome profiling data (Han et al. 2020) to ask if collision-prone sequences were enriched in ESMs relative to other transcripts. Comparing the abundance of disomes in ESMs relative to monosomes revealed a modest enrichment relative to OSLs or all transcripts (Fig. 3E). These data suggest that there are higher levels of collisions in these messages, even in untreated cells.

### mRNA stabilization is independent of ribosome collisions

**FIGURE 4.**
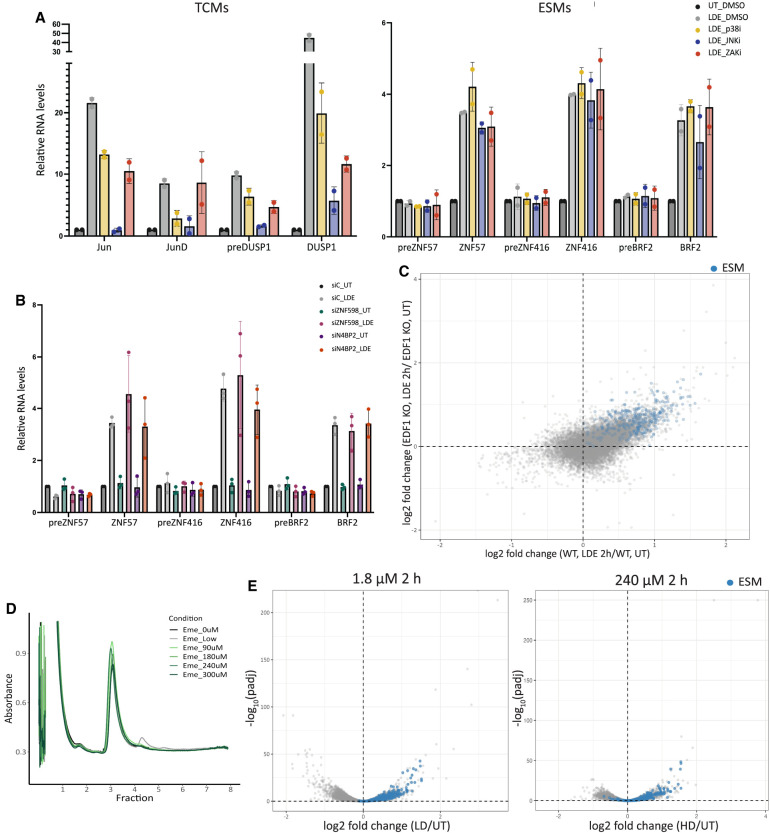
Stabilization of select transcripts is independent of ribosome collisions. (*A*) RT-qPCR data from cells preincubated with inhibitors against RSR-activated signaling pathways obtained using primers against ESMs (*right*) or emetine-induced TCMs (*left*). (*B*) RT-qPCR data obtained using primers against ESMs in untreated samples or emetine-treated samples transfected with siRNAs against ZNF598 (siZNF598), N4BP2 (siN4BP2), or with a nontargeting control (siC) (*n* = 3). (*C*) Scatter plot showing the log_2_(fold change) of emetine-treated versus untreated EDF1 KO cells on the *y*-axis and the log_2_(fold change) of wild-type (WT) emetine-treated versus untreated HEK293 Flp-In TRex cells on the *x*-axis (Sinha et al. 2020). ESMs are highlighted in blue. (*D*) Polysome traces obtained from RNase A digested lysates from cells treated with increasing emetine concentrations showing the A260 absorbance on the *y*-axis and the sucrose gradient fraction number on the *x*-axis. (*E*) Volcano plots for RNA-seq data from low-dose (LD, *left*) or high-dose (HD, *right*) emetine-treated samples showing the −log_10_(adjusted *P*-value) on the *y*-axis and the log_2_(fold change) of emetine-treated versus untreated on the *x*-axis.

We next sought to investigate a potential role for ribosome collisions in emetine-induced mRNA stabilization. Because low doses of emetine lead to ribosome collisions (Supplemental Fig. S1A,B; Juszkiewicz et al. 2018; Sinha et al. 2020), we tested whether the mRNA stabilization of the ESMs that we observe was dependent on collisions and the subsequent activation of RSR-mediated signaling pathways. We assessed RSR activation by using RT-qPCR to measure the levels of transcripts that are known to be transcriptionally upregulated upon ZAKα activation. As expected, pretreatment of cells with inhibitors of ZAKα, p38, or JNK led to a decrease in the synthesis-driven upregulation of *JUN, JUND,* and *DUSP1* upon 2 h emetine treatment compared to a control that was pretreated with DMSO (Iordanov et al. 1997; Ouyang et al. 2005; Sinha et al. 2020; Wu et al. 2020). However, these inhibitors did not affect the levels of ESMs (Fig. 4A), suggesting that stabilization of these mRNAs is independent of RSR activation.

Recent work argues that translation of C2H2 ZNF-encoding transcripts results in ribosome collisions (even in untreated cells) leading to ZNF598-mediated mRNA decay (Ishibashi et al. 2024). Since we observe an enrichment of disomes on ESMs, we reasoned that inducing widespread ribosome collisions with emetine might overwhelm ZNF598, leading to a decrease in the decay of its canonical targets. At the same time, we wanted to assess the importance of the putative collision-dependent endonuclease N4BP2, homologous to the yeast endonuclease Cue2 (D'Orazio et al. 2019) and to NONU-1 in *Caenorhabditis elegans* (Glover et al. 2020), in regulating the levels of these transcripts. siRNA knockdowns of ZNF598 or N4BP2 did not change the levels of ESMs in untreated or emetine-treated cells (Fig. 4B; Supplemental S5A). Additionally, analysis of publicly available RNA-seq data from untreated cells lacking ZNF598 or the translational repressor that is recruited to collided ribosomes by EDF1, GIGYF2, did not show an increase in the levels of ESMs (Supplemental Fig. S5B,C; Hickey et al. 2020). Moreover, analysis of RNA-seq data obtained from EDF1 knockout (KO) cells treated with emetine for 2 h (Sinha et al. 2020) did not reveal any significant changes in the levels of ESMs (Fig. 4C; Supplemental Fig. S5D). Together, these results argue that factors that recognize and resolve ribosome collisions are not responsible for the observed emetine-induced stabilization of this set of transcripts.

Finally, we evaluated the potential importance of ribosome collisions for transcript stabilization by using higher doses of emetine that stall ribosomes, but do not lead to collisions. Emetine doses were titrated, and collisions were measured by assessing the presence of nuclease-resistant disomes. Emetine concentrations as low as 90 µM resulted in no visible accumulation of collided disomes (Fig. 4D), as well as a lack of eS10 ubiquitylation (Supplemental Fig. S5E). We then assessed the collision dependence of transcript stabilization genome-wide by performing RNA-seq on cells treated with 1.8 µM emetine (low dose) or 240 µM emetine (high dose) for 2 h.

RNA-seq analysis shows that levels of most stabilized transcripts also increase at high doses (Fig. 4E), revealing that stabilization is independent of the concentration of emetine used. Consistent with this idea, RT-qPCR data obtained from the emetine titration showed an increase in the levels of the stabilized transcripts at all doses, while RT-qPCR using primers against intronic regions of these transcripts showed no changes to the transcription of the stabilized mRNAs regardless of the dose of emetine used (Supplemental Fig. S5F). Finally, we tested whether stabilization of these transcripts could be reproduced with a different translation inhibitor, anisomycin. RT-qPCR data for several of the ESMs following anisomycin treatment revealed a mild dose-independent increase in levels of the tested transcripts (Supplemental Fig. S5G). Together, these data demonstrate that stabilization of these mRNAs is induced by translation inhibition independent of ribosome collisions.

### Concluding remarks

Here, we explored the impact of translation elongation inhibitors on the transcriptome. By performing TL-seq on cells treated with low doses of emetine, we identify a group of mRNAs that are strongly stabilized upon emetine treatment (termed ESMs). It is worth noting that, upon short s^4^U incubations like the ones performed in this study, the TL-seq method is more sensitive to changes in transcripts with higher turnover rates, as the rate of s^4^U incorporation scales with the transcription and decay rates of each transcript at steady state. Consistent with this idea, we find that ESMs have short half-lives, and that this characteristic correlates with the observed emetine-induced stabilization but does not explain why ESMs are stabilized while OSLs are not.

The ESMs predominantly encode a class of proteins with zinc finger domains, which are frequently found in transcription factors. Zinc finger domain-encoding genes are rapidly evolving and represent a large portion of the genome (Wells et al. 2023). The transcripts encoding this class of genes are enriched in suboptimal codons (Diez et al. 2022). The fact that these genes are rapidly evolving might explain their low abundance as their expression may not yet have been optimized. Consistent with the high number of C2H2 ZNF-encoding mRNAs in ESMs, we find that ESMs have a high frequency of suboptimal codons compared to other expressed mRNAs. Interestingly, we find that ESMs encoding C2H2 ZNFs have a greater enrichment of suboptimal codons than other C2H2 ZNF-encoding transcripts, suggesting that codon optimality is a contributor to emetine-induced mRNA stabilization. However, this feature does not fully explain the short half-lives of ESMs since other mRNAs with a higher frequency of suboptimal codons have longer half-lives.

Importantly, using low or high doses of emetine and anisomycin to inhibit translation, we observe that mRNA stabilization occurs both upon ribosome stalling and upon ribosome collisions. The increase in the levels of stabilized transcripts observed after anisomycin treatment were lower than the levels observed upon emetine treatment. Possible differences between ribosome conformations induced by these different drugs could impact the recruitment of degradation machinery to the mRNAs trapped with stalled or collided ribosomes resulting in differences in decay efficiencies. For example, the availability of the E site on elongating ribosomes may be critical for the normal degradation of the ESMs (Buschauer et al. 2020; D'Orazio and Green 2021), and this may be more effectively occluded on emetine relative to anisomycin stalled ribosomes.

Because induction of ribosome collisions leads to the recruitment of many different QC factors, including those driving mRNA decay (D'Orazio and Green 2021), we were surprised to observe a decrease in the rate of degradation of many transcripts in conditions that induce ribosome collisions (D'Orazio et al. 2019; Saito et al. 2022). We initially reasoned that this might be because the induction of collisions would titrate these QC factors away from their canonical targets, which would include ESMs. However, the knockdown of putative mammalian nucleases or other factors implicated in this process had no effect on ESM levels in untreated or emetine-treated cells. These data argue against collision-induced regulation of these transcripts in untreated cells as proposed (Ishibashi et al. 2024).

Our results reveal a class of mRNAs whose degradation is particularly sensitive to the inhibition of translation elongation. Based on their short half-lives and their enrichment for suboptimal codons, we favor a model in which their decreased degradation rates are facilitated by a decrease in cotranslational mRNA decay, independent of ribosome collisions. Stalled or collided ribosomes could limit the access of decay factors such as Xrn1, the CCR4-NOT complex, or the exosome to the trapped mRNAs. While we did not observe increased levels of ESMs after knocking down components of the CCR4-NOT complex, it is possible that these knockdowns are not sufficient to prevent the degradation of these mRNAs by any remaining CCR4-NOT complex. However, it is also possible that the decay of ESMs relies on different decay machinery or that there is redundancy in the pathways that regulate the decay of these mRNAs. Moreover, while the data presented here supports a CDS-mediated decay mechanism, we cannot discard the possibility that UTRs contribute to the emetine-induced stabilization of ESMs. Defining the mechanisms that selectively impede the decay of ESMs, but not OSLs, upon translation elongation inhibition will further our understanding of the connections between translation and mRNA metabolism.

## MATERIALS AND METHODS

### Cell culture

Experiments were performed in HEK-293T cells purchased from ATCC. HEK-293Ts were cultured in DMEM (Thermo Fisher) supplemented with 10% fetal bovine serum (Thermo Fisher) and kept in a 37°C humidified incubator at 5% CO_2_. For emetine treatments, cells were seeded at a density of 2 × 10^6^ in 10 cm dishes 48 h before the experiment. On the day of treatment, the media were changed 2 h before the addition of translation inhibitors. Aqueous emetine from 100 µM stocks was added to treated cells at 1.8 µM for low doses and at 240 µM for high doses, unless otherwise stated.

### siRNA knockdowns

Cells were seeded at a density of 0.15 × 10^6^ in 6-well plates and transfected using 50 nM of siRNAs 24 h after plating using Lipofectamine RNAiMAX in OPTI-MEM (Thermo Fisher), according to manufacturer's instructions. Transfected cells were incubated with siRNAs for an additional 48 h, after which the media were changed 1 h before any additional treatments.

### RNA extractions and RT-qPCR

RNA was extracted using TRIzol (Thermo Fisher) according to manufacturer's instructions. DNase treatments were performed by incubating 10 µg of RNA with 0.4 units of TURBO DNase (Thermo Fisher) per microliter in 200 µL reactions at 37°C for 30 min. RNAs were recovered by performing a Phenol:Chloroform:Isoamyl cleanup followed by an ethanol precipitation. For experiments where RNA was extracted from sucrose gradient fractions, 700 µL of each fraction was added to the Maxwell RSC miRNA Tissue Kit (Promega) and processed using the Maxwell instrument. For reverse transcription, the iScript cDNA Kit (Bio-Rad) was used according to manufacturer's instructions. cDNAs obtained from whole cell lysates were diluted 1:4, while cDNAs obtained from gradient fractions were diluted 1:1 in water before proceeding to qPCR. qPCRs were performed using iTaq Universal SYBR Green Supermix (Bio-Rad) according to manufacturer's instructions. RT-qPCR data were analyzed using the ddCt method using the geomean for 18S and U2 as a control (Schmittgen and Livak 2008). For Actinomycin D and RT-qPCR across sucrose gradient fractions, 50 pg of eGFP mRNA was added before TRIzol extractions and used as a spike-in control for downstream normalization (Table 1).

**TABLE 1.** Oligos used in this study

Target	Forward	Reverse	Source
ZNF57	CTGGCCTCAGTAGATGATGGG	TGTGAAGTTTGGTATTGTCTGTTCC	This study
preZNF57	GCAGAGTTTGCGACAGTGTG	AACCAGAATGGAACACGCCT	This study
ZNF416	GGATTCGACTTCGGTTCCCG	AAGGAGCCCCCATTCTTCCT	This study
preZNF416	GCACTCTTTGGGTCCTTGGT	TGAGACAACGTTCCACACCT	This study
BRF2	GGAAGACTCGCACTATTCGCA	TCGTTTTCCCCTGTGCTTCG	This study
preBRF2	TCAGATTCCTTTTGCCTCCCC	TGCCCGATACTAAGTGCTGG	This study
MAT2A	CTGCTCCTTCGTAAGGCCAC	TGGTCACAAATCTTATCTGGGTG	This study
NETO2	TGCTCGGTCCTCAAAGTGTT	GAACCCAAATGCCACACTGG	This study
RPL35	CGAGTCGTCCGGAAATCCAT	GGTCCAGGGGCTTGTACTTC	This study
preRPL35	TCAAGTGTTGGTGGGAAGCG	CCGTTGTGTGCTTAGCCTCT	This study
GAPDH	CTGCACCACCAACTGCTTAG	GTCTTCTGGGTGGCAGTGAT	Vilborg et al. (2015)
preGAPDH	AGATTTGGTCGTATTGGGCG	CTCACCATGTAGCACTCACC	This study
JUN	GAGCTGGAGCGCCTGATAAT	CCCTCCTGCTCATCTGTCAC	This study
JUND	CGTTGTCGCCCATCGACAT	TTTCTCTTCCAGGCGCGAGA	This study
preDUSP1	AGAACTGGCAAAGGCATGGA	CGGCTCCGTCAGACCACTTA	This study
DUSP1	GGCCATTGACTTCATAGACTCCA	AACTCAAAGGCCTCGTCCAG	This study
U2	CTCGGCCTTTTGGCTAAGAT	TATTCCATCTCCCTGCTCCA	Vilborg et al. (2015)
18S	CGAAAGCATTTGCCAAGAAT	GCATCGTTTATGGTCGGAAC	Vilborg et al. (2015)
eGFP	CCCGACAACCACTACCTGAG	GTCCATGCCGAGAGTGATCC	This study

### Sucrose gradient sedimentation

After treatment, cells were quickly rinsed once with 4 mL of 1× PBS supplemented with 360 µM emetine to freeze ribosomes and prevent run-off. Once PBS was removed, 250 µLs of lysis buffer [50 mM HEPES pH 7.4, 100 mM KOAc, 5% glycerol, 0.5% Triton X, 15 mM Mg(OAc)_2_, 360 µM emetine, 1× Halt Protease and Phosphatase Inhibitor (Thermo Fisher), 80 units of TURBO DNase, 1 mM TCEP, 200 units of SUPERase In (Thermo Fisher)] were added to the plate, cells were scraped, transferred to a 1.5 mL tube, flash frozen using liquid nitrogen and stored at −80°C. Thawed lysates were normalized to RNA amounts using the Qubit High Sensitivity Kit (Invitrogen) to ensure even loading of gradients. Sucrose gradients were made using 6 mL of 10% and 6 mL of 50% sucrose containing 1× sucrose gradient buffer [250 mM HEPES pH 7.5, 1 M KOAc, 50 mM Mg(OAc)_2_] and prepared using a gradient master (Biocomp). After loading ≥ 30 µg of RNA in a total volume of 350 µL, gradients were balanced and spun at 40,000 rpm for 1.5 h (for RT-qPCR) or for 1.75 h at 4°C in an ultracentrifuge using a Beckman SW-41 rotor. Gradients were fractionated using a Biocomp Piston Gradient Fractionator with simultaneous measurement of the UV absorbance (260 nm) across the gradient. For immunoblotting, protein samples were recovered by adding TCA to each fraction to a final concentration of 16%, incubated at −20°C overnight, and precipitated by centrifugation (21,100*g*, 4°C for 30 min). Pellets were rinsed twice using 100% acetone and dried using a vacuum evaporator at 42°C for 3 min. Dried pellets were resuspended in 30 µL of 6× Laemmli buffer, supplemented with 10 µL of Tris pH 8.0 and boiled for 10 min at 95°C, 1000 rpm. After boiling, 80 µL of water was added to each fraction.

### Immunoblotting

Proteins from whole cell lysates or gradient fractions were loaded onto a 4%–20% Criterion TGX Precast Midi Protein Gel (Bio-Rad) and run at 120 volts for 1.5 h. Afterward, proteins were transferred onto a nitrocellulose membrane using the Trans-Blot Turbo RTA Midi Nitrocellulose Transfer Kit (Bio-Rad) in a Trans-Blot Turbo Transfer System using the TURBO protocol for one midigel. Membranes were blocked for ≥ 1 h with 5% milk dissolved in 1× TBST, or with Intercept (TBS) Blocking Buffer for LI-COR imaging. Afterward, primary antibodies were diluted in 5% milk in 1× TBST or in Intercept T20 (TBS) Antibody Diluent for LI-COR imaging, and membranes were incubated overnight at 4°C (Table 2). Corresponding secondary antibodies (LI-COR: IRDye 680RD Donkey anti-Rabbit IgG; Chemiluminescence: Mouse anti-rabbit IgG-HRP [sc2357] or anti-mouse IgG, HRP-linked Antibody [CST #7076]) were diluted 1:2000 in 5% milk in 1× TBST or in Intercept T20 (TBS) Antibody Diluent for LI-COR imaging and added to membranes for ≥1 h. Blots for proteins isolated from gradient fractions were visualized using the LI-COR system according to standard protocol. Blots for proteins from whole cell lysates were imaged using a 1:10 dilution of SuperSignal West Femto Maximum Sensitivity Substrate in SuperSignal West Pico PLUS chemiluminescent substrate.

**TABLE 2.** Primary antibodies used in this study

Target	Brand	Catalog no.	Dilution
CNOT3	Abnova	H00004849-M01	1:800
CNOT4	Abcam	ab214937	1:800
N4BP2	Abnova	H00055728-A01	1:400
ZNF598	Bethyl Laboratories	A305-108A	1:1000
Vinculin	Santa Cruz	sc-73614	1:2000
EDF1	Abcam	ab174651	1:800
uS3	Abcam	ab140688	1:2000
uS10	LSBio	LS‐C335612	1:2000

### Time lapse sequencing

For TL-seq, cells were incubated with 500 µM s^4^U for 1 h with or without 1.8 µM emetine. After treatment, cells were scraped in 1× PBS, spun down at 350*g* for 3 min, and resuspended in lysis buffer. In total, 100 µL of lysate was added to 1 mL of TRIzol. After RNA extraction, RNAs treated with TURBO DNase were recovered using RNA XP beads (Beckman). RNAs were then subjected to TimeLapse chemistry (Schofield et al. 2018) by incubating samples at 45°C for 1 h with 600 mM TFEA, 1 mM EDTA, 100 mM sodium acetate pH 5.2, and 10 mM sodium periodate, and then reduced by incubating samples at 37°C for 30 min with 100 mM DTT, 100 mM Tris pH 7.4, 10 mM EDTA, and 1 mM NaCl. After each step, samples were recovered using RNA XP beads. Samples were kept in the dark throughout this process. Libraries were prepared using the TruSeq Stranded mRNA Library Prep Kit (Illumina) and sequenced on the Nova-Seq platform by the Single Cell and Transcriptomics Core at Johns Hopkins University School of Medicine.

### RNA sequencing

RNA sequencing libraries prepared for cells treated with high or low doses of emetine for 2 h were generated as described above. For differential expression analyses, normalization factors were generated from reads corresponding to ERCC spike-in controls (Lexogen SIRV Set 4, catalog 141.0*) that were added based on cell count, according to the manufacturer's instructions. The SMARTer Stranded Total RNA Sample Prep Kit - HI Mammalian Kit was used to prepare libraries, which were then sequenced on the Nova-Seq platform by the Single Cell and Transcriptomics Core at Johns Hopkins University School of Medicine.

### Data analyses

Adapter reads were removed using cutadapt ‐‐minimum-length = 20 -q 20,20 (version 1.9.1). Reads were mapped to the hg38 genome using hisat-3n (version 2.2.1-3n-0.0.2) ‐‐base-change T,C. Samtools was used to generate, sort, and index bam files. For standard RNA-seq analyses, counts were generated using htseq-count (version 0.13.5). For TL-seq, bam files were processed using bam2bakR, which also generated the tdf files used to create genome browser images (https://github.com/simonlabcode/bam2bakR). DESeq2 (version 1.38.3) was used for differential expression analysis of transcripts with over 100 read counts, and bakR was used for kinetic analysis of TL-seq data. Mechanism scores for ESMs and TCMs were generated using a hybrid fit and the NSSheat command on bakR, while mechanism scores for nondifferentially expressed transcripts were generated using DissectMechanism. Gene ontology analyses for protein domains were done using DAVID. Mean half-life calculations were obtained from Schofield et al. (2018). CSC analyses were performed on calculations from Wu et al. (2019) using a custom script and CDS sequences from BioMart. TE calculations were generated using data from Han et al. (2020). Statistical analyses for CSC and TE data were performed using Kolmogorov–Smirnov tests.

## DATA DEPOSITION

High-throughput sequencing data generated for this study are available under the NCBI GEO series GSE264661.

## SUPPLEMENTAL MATERIAL

Supplemental material is available for this article.

## Figures and Tables

**Nicolle Rosa-Mercado:**
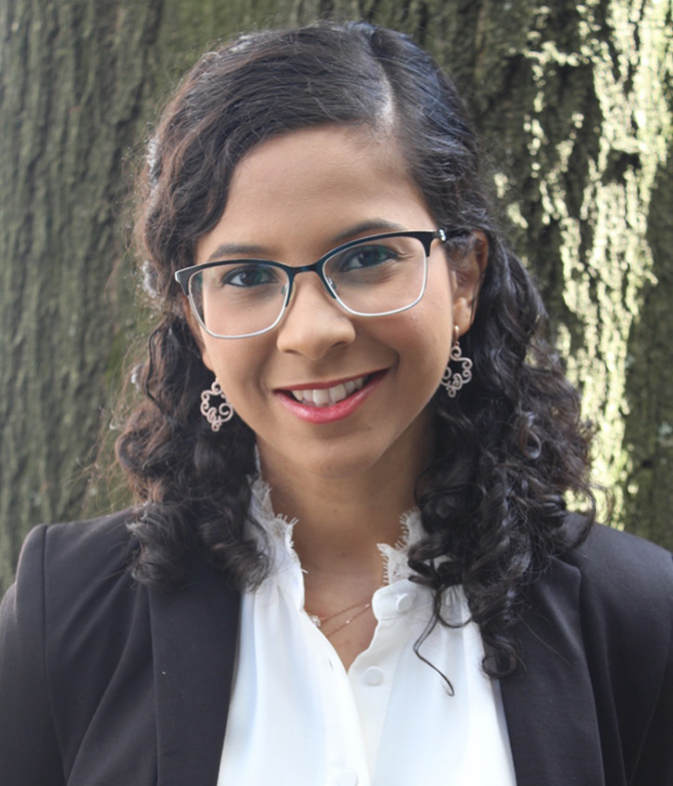


## MEET THE FIRST AUTHOR

***Meet the First Author(s)* is an editorial feature within *RNA*, in which the first author(s) of research-based papers in each issue have the opportunity to introduce themselves and their work to readers of *RNA* and the RNA research community. Nicolle Rosa-Mercado is the first author of this paper, “Translation elongation inhibitors stabilize select short-lived transcripts.” Nicolle is an HHMI Hanna Gray fellow in Rachel Green's lab at Johns Hopkins University School of Medicine, where she studies the interplay between mRNA dynamics and translational regulation.**

**What are the major results described in your paper and how do they impact this branch of the field?**

In our manuscript, we characterize a class of mRNAs that are stabilized upon treatment of human cells with the translation elongation inhibitor, emetine. We find that these transcripts are short-lived and enriched in suboptimal codons, but that these two characteristics are independent from one another. Moreover, these emetine-stabilized mRNAs encode many C2H2 zinc finger proteins. Emetine is commonly used to study different aspects of molecular biology. Our results provide a detailed understanding about how this drug reshapes the transcriptome.

**What led you to study RNA or this aspect of RNA science?**

During graduate school, I became interested in how different stress conditions affect RNA metabolism. Previous work from the Green lab provided a fundamental understanding of how stress affects ribosomes. When I joined the lab, I decided to combine my interest in RNA dynamics with the lab's expertise in studying ribosome distress.

**During the course of these experiments, were there any surprising results or particular difficulties that altered your thinking and subsequent focus?**

Our group and others have shown that defects in translation often lead to the degradation of the affected transcript. Therefore, we started this project expecting to find an increase in the turnover of many transcripts upon emetine treatment. We were very surprised to instead find a group of mRNAs that are stabilized by this treatment and became eager to characterize these.

**Are there specific individuals or groups who have influenced your philosophy or approach to science?**

My thesis advisor, Joan Steitz, greatly influenced the way that I approach scientific questions, but more importantly, she taught me by example to be an outstanding member of the scientific community. My scientific philosophy was also shaped by my undergraduate research mentors, Robert Ross and Michael Rubin from the University of Puerto Rico at Cayey, who provided me with research opportunities on and off campus.
